# Reduced SOD2 expression does not influence prion disease course or pathology in mice

**DOI:** 10.1371/journal.pone.0259597

**Published:** 2021-11-04

**Authors:** Simote T. Foliaki, Brent Race, Katie Williams, Chase Baune, Bradley R. Groveman, Cathryn L. Haigh

**Affiliations:** 1 Prion Cell Biology Unit, Laboratory of Persistent Viral Diseases, Rocky Mountain Laboratories, National Institute of Allergy and Infectious Diseases, National Institutes of Health, Hamilton, MT, United States of America; 2 Veterinary Biology Unit, Laboratory of Persistent Viral Diseases, Rocky Mountain Laboratories, National Institute of Allergy and Infectious Diseases, National Institutes of Health, Hamilton, MT, United States of America; University of Verona, ITALY

## Abstract

Prion diseases are progressive, neurodegenerative diseases affecting humans and animals. Also known as the transmissible spongiform encephalopathies, for the hallmark spongiform change seen in the brain, these diseases manifest increased oxidative damage early in disease and changes in antioxidant enzymes in terminal brain tissue. Superoxide dismutase 2 (SOD2) is an antioxidant enzyme that is critical for life. SOD2 knock-out mice can only be kept alive for several weeks post-birth and only with antioxidant therapy. However, this results in the development of a spongiform encephalopathy. Consequently, we hypothesized that reduced levels of SOD2 may accelerate prion disease progression and play a critical role in the formation of spongiform change. Using SOD2 heterozygous knock-out and litter mate wild-type controls, we examined neuronal long-term potentiation, disease duration, pathology, and degree of spongiform change in mice infected with three strains of mouse adapted scrapie. No influence of the reduced SOD2 expression was observed in any parameter measured for any strain. We conclude that changes relating to SOD2 during prion disease are most likely secondary to the disease processes causing toxicity and do not influence the development of spongiform pathology.

## Introduction

The transmissible spongiform encephalopathies, or prion diseases (PrDs), are a family of fatal, transmissible neurodegenerative conditions. Characteristic features of prion diseases include deposition of abnormal prion protein within the brain, astrogliosis and spongiform vacuolation. The causative agents (prions) arise from mis-folding of a normal cellular protein, the prion protein (PrP), into disease-associated conformers that can then template further mis-folding and propagation of prions. Various indications of damage are found in terminal brain tissue, including signs of substantial oxidative stress [1, 2].

Reactive oxygen and nitrogen species (referred to collectively herein as ROS) are produced as byproducts of various cellular reactions and are known to have critical roles as cellular signaling molecules. Oxidative damage occurs when the mechanisms producing ROS exceed cellular detoxifying capacity. Markers of oxidative damage within the brains of people dying from PrD include lipid peroxidation and protein nitration [2]. It is unclear whether oxidative damage plays a causative role in the disease pathogenesis or whether it is a consequence of failing detoxification pathways. There are various enzyme systems responsible for the detoxification of ROS, including the superoxide dismutase (SOD) family. The SOD family comprises three members; SOD1 or CuZnSOD, located ubiquitously throughout the cell cytoplasm and mitochondrial intermembrane space, SOD2 or MnSOD, confined to the mitochondrial matrix, and SOD3 or extracellular SOD found in the extracellular matrix [3]. Failure of one of these enzymes to adequately detoxify ROS could permit oxidative damage to accumulate to toxic levels.

Animal models have been used to consider the role of ROS in PrD and the importance of cellular antioxidant defenses. It has been shown that lipid peroxidation begins early during the course of murine prion infection, around the time of initial deposition of PrP and spongiform development, well before onset of symptoms [4]. Reduction of SOD1 protein in mouse models of PrD accelerates disease [5], however, the role of SOD2 in prion infection was, until now, un-reported. SOD2 expression is essential for life and, in its absence, mice die perinatally of dilated cardiomyopathy [6]. However, if SOD2 knock-out mice are kept alive for several weeks post birth using antioxidant therapy they develop a spongiform encephalopathy reminiscent of a PrD [7]. At the terminal phase of murine PrD SOD activity [8, 9] and SOD2 expression [10, 11] were previously found to be decreased. In support of a loss of function of SOD2 during disease, a SOD2 mimetic has been shown to significantly extend the lifespan of prion infected mice and reduce spongiform vacuolation [12]. Additionally, in cell culture systems modelling prion infection, increased ROS production and damage to cellular proteins and lipids have been found [13]. Of interest, within this system, reduction of SOD2 protein was associated with cytosolic localization and degradation by caspases [14]. Caspase activity is increased as mice approach terminal PrD [15, 16] and caspase 3 has directly been shown to cleave SOD2 [17], therefore an increased turnover and resultant loss of function of SOD2 could be involved in disease progression or formation of spongiform damage.

Potentially, if PrP influences SOD2 function and this function is critical for maintaining homeostasis during prion disease, its loss toward terminal disease could be responsible for the accumulating cellular damage including development of spongiform change. It is not possible to consider the influence of SOD2 by investigating disease in SOD2 knock-out mice due to their short lifespan. However, SOD2 heterozygous mice, which express approximately 50% of the SOD2 protein levels of wild type mice, develop normally and have a normal life expectancy with only a few reported effects of the reduced protein levels [18–23]. The SOD2 heterozygous mice do show a clinically silent underlying mitochondrial oxidative stress [24, 25], which renders them more susceptible to oxidative insults, such as what may occur during prion infection. We hypothesized that if SOD2 is critical for maintaining homeostasis and that this fails during disease, the reduction of SOD2 in SOD2 heterozygous knock-out mice should be sufficient to accelerate the disease course and exacerbate the spongiform change. Our results indicate that SOD2 reduction does not influence acute or long-term responses to three different scrapie prion strains.

## Methods

### Animal ethics statement

All mice were housed at the Rocky Mountain Laboratory (RML) in an AAALAC-accredited facility in compliance with guidelines provided by the Guide for the Care and Use of Laboratory Animals (Institute for Laboratory Animal Research Council). All experiments were approved by the RML Animal Care and Use Committee, protocol 2018-072-E. Mice were socially housed in groups of 3–4 mice per box in climate controlled disposable caging with wood shavings and nestlet bedding provided for enrichment. Room temperatures were kept at 70–72˚F and a 12-hr light-dark cycle was used. Mice had free access to food and water.

### Mice

Heterozygous B6.129S7-Sod2tmLeb/J^(+/-)^ (Jackson Laboratories) were crossed to produce B6.129S7-Sod2tmLeb/J^(+/-)^ (SOD2^+/-^) and Sod2tmLeb/J^(+/+)^ (WT) littermate wild type controls. Genotypes were confirmed by PCR analysis as described by Jackson Laboratory. Primers detecting the mutant, TGT TCT CCT CTT CCT CAT CTC C (oIMR0781) and ACC CTT TCC AAA TCC TCA GC (oIMR0782) along with wild type primers, TGA ACC AGT TGT GTT GTC AGG (oIMR0878) and TCC ATC ACT GGT CAC TAG CC (oIMR0888) were used to distinguish genotypes of littermate controls.

### Infections

Prior to experimentation we used Graphpad Statmate software to determine reasonable group sizes to detect an estimated difference in incubation periods of 1 week. Groups of 10–12 male mice per genotype (total = 69 mice) were intracerebrally inoculated with 30 μl of a 1% (w/v) brain homogenate from 22L, RML or ME7 prion strains. Mice were anesthetized with isoflurane and inoculations were performed using a ½ inch long, 27-gauge needle on a 1 mL slip tip syringe. The inoculum was injected into the left cerebral hemisphere, approximately 2 mm lateral to the midline and 2–3 mm deep, near the region of the thalamus. The titers of these scrapie stocks had been determined previously in C57 mice and contained the following 50% infective doses (ID_50_) in each 30-μl volume: 22L, 6.0 × 10^5^; RML, 2.4 × 10^4^; ME7, 5.0 × 10^4^. Normal control mice were either ‘mock’ inoculated with uninfected brain homogenates (N = 4) or not inoculated (N = 4). Mice were monitored by evaluators blind to genotype twice weekly prior to onset of clinical signs, then every 1–3 days throughout the clinical phase (where they begin showing decrease in body condition, increased somnolence and kyphosis) and euthanized by isoflurane inhalation overdose followed by cervical dislocation when they developed terminal clinical signs (signs include ataxia, tremors, kyphosis, hyperactivity, somnolence, poor grooming, reduction in body condition). One SOD2^+/-^ mouse from the 22L experiment was excluded from analysis. The mouse was euthanized at 67 dpi for unresolving dermatitis. This time point is far prior to any clinical signs of prion disease. Log-rank (Mantel-Cox) statistical analysis was performed for each prion strain using GraphPad Prism 8 software.

### H&E staining, immunohistochemistry and quantification

Brain fixation, paraffin embedding, histology and immunohistochemistry for prion protein (antibody D13) and anti-GFAP were carried out as described previously [26]. Positive pixel quantification was performed on brain sections from 5–6 mice per experimental group for D13 (produced in house as described in [27]) and GFAP stained sections using the ImageScope positive pixel count algorithm (version 9.1) as described [28] with the following modification. In the current manuscript, all positive pixels (including weak, moderate and strong) were included for the D13 analysis.

### Lesion profiling

Lesion profiling was completed on 5–6 mice from each genotype from the 22L and ME7 infection experiments. Sagittal brain sections were cut approximately 0.5 mm from midline and stained by routine H&E protocols. Each section was scored for the severity of spongiform vacuolar degeneration in 4 gray matter areas (cerebral cortex, thalamus, superior colliculus and cerebellum). Spongiosis was scored as follows: 0, no vacuoles; 1, few vacuoles widely and unevenly distributed; 2, few vacuoles evenly distributed; 3, moderate numbers of vacuoles evenly distributed; and 4, many vacuoles with some confluences. An average score and standard deviation for each experimental group was calculated and shown in Fig 6B.

### Western blotting

10% (w/v) brain homogenates were prepared in 1x PBS, further diluted into 1% (w/v) for western blotting analysis. Proteins were denatured by boiling for 5 minutes in 1× sample buffer (containing 6% Beta-mercaptoethanol), resolved in Bolt 4–12% Bis-Tris Plus gels (Invitrogen), and transferred to PVDF membrane (Millipore). SeeBlue Pre-stained Protein Standard (Life Technologies) was resolved alongside the protein samples. The membrane was incubated in blocking solution (5% w/v non-fat milk in 1× TBS and 0.05% Tweens) for 1hr at room temperature and in anti-SOD2 antibody (1:5000 dilution in blocking solution; Abcam: ab13533), anti-SOD1 (1:4000 dilution in blocking solution; Abcam: ab13498) or anti-SOD3 (1:4000 dilution in blocking solution; Abcam: ab83108) overnight at 4°C. The primary antibody was coupled with the appropriate secondary antibody (HRP conjugate), and the protein bands were visualized using ECL Select (Amersham) and imaged by iBright imaging system (Invitrogen). Full blot images are shown in Sup File 1.

### Proteinase K digestion and PrP immunoblotting blotting

10% (w/v) brain homogenates in PBS were digested with Proteinase K, run on 12% tris-glycine gels and transferred to PBDF membranes as previously described [27]. Immunoblots were probed as described [27] with the two following modifications, anti-PrP antibody D13 was used at a 1:1,000 dilution and the secondary antibody (anti-human) was used at a 1:20,000 dilution.

### Electrophysiology

As described previously [29], 300 μm thick hippocampal sections were collected from 12-week-old mice in an ice-cold cutting solution (3 mM KCl, 25 mM NaHCO_3_, 1.25 mM NaH_2_PO_4_, 206 mM Sucrose, 10.6 mM Glucose, 6 mM MgCl_2_.6H_2_O, 0.5 mM CaCl_2_.2H_2_O) using a vibratome (Leica VT1200S). The hippocampal slices were incubated for 1 hr at 32°C in carbogenated (5% CO_2_; 95% O_2_) artificial cerebrospinal fluid (aCSF: 126 mM NaCl, 2.5 mM KCl, 26 mM NaHCO_3_, 1.25 mM NaH_2_PO_4_, 10 mM Glucose, 1.3 mM MgCl_2_.6H_2_O and 2.4 mM CaCl_2_.2H_2_O). The slices were mounted on to 60MEA200/30iR-Ti-pr-T multi-electrode arrays (MEA; Multichannel Systems; Germany) for the electrophysiological recording while being continuously superfused with carbogenated aCSF. The Shäffer collateral pathway was stimulated by electric stimulation (2000–2500 mV) to evoke the field excitatory post-synaptic potential (fEPSP) at the CA1 region. The baseline stimulation intensity was determined by an input-output curve obtained from stimulating the pathway with increasing strength of electric stimulation (input), starting at 500 mV to a stimulation intensity (4000–5000 mV) that evoked the maximum fEPSP amplitude (output). This max response was indicated by the point where the input-output curve reached a plateau. The stimulation strength that evoked 30–50% of the maximum fEPSP amplitude was the baseline stimulation intensity. The baseline fEPSP was recorded for 30 minutes by applying the baseline stimulation intensity in 30 seconds interval. Following the first five-minute baseline, the slices were treated with 0.5% (w/v) brain extracts (normal brain homogenates or PrPSc-infected mouse brain homogenates in aCSF) for 10 minutes. The 0.5% (w/v) dosage was determined based on characterizations done for previous studies, we chose this dose after determining that higher concentrations caused non-specific tissue damage [29]. After this treatment, the superfusion returned to aCSF for the rest of the recording. After the baseline recording, the LTP was induced by Tetani, consisting of three 100 Hz trains (500 ms long per train) delivered in 20 sec interval. The post-tetani fEPSPs were recorded for 30 minutes, with the last ten-minute recording was averaged and used for the statistical analysis of LTP.

## Results

**Fig 1:**
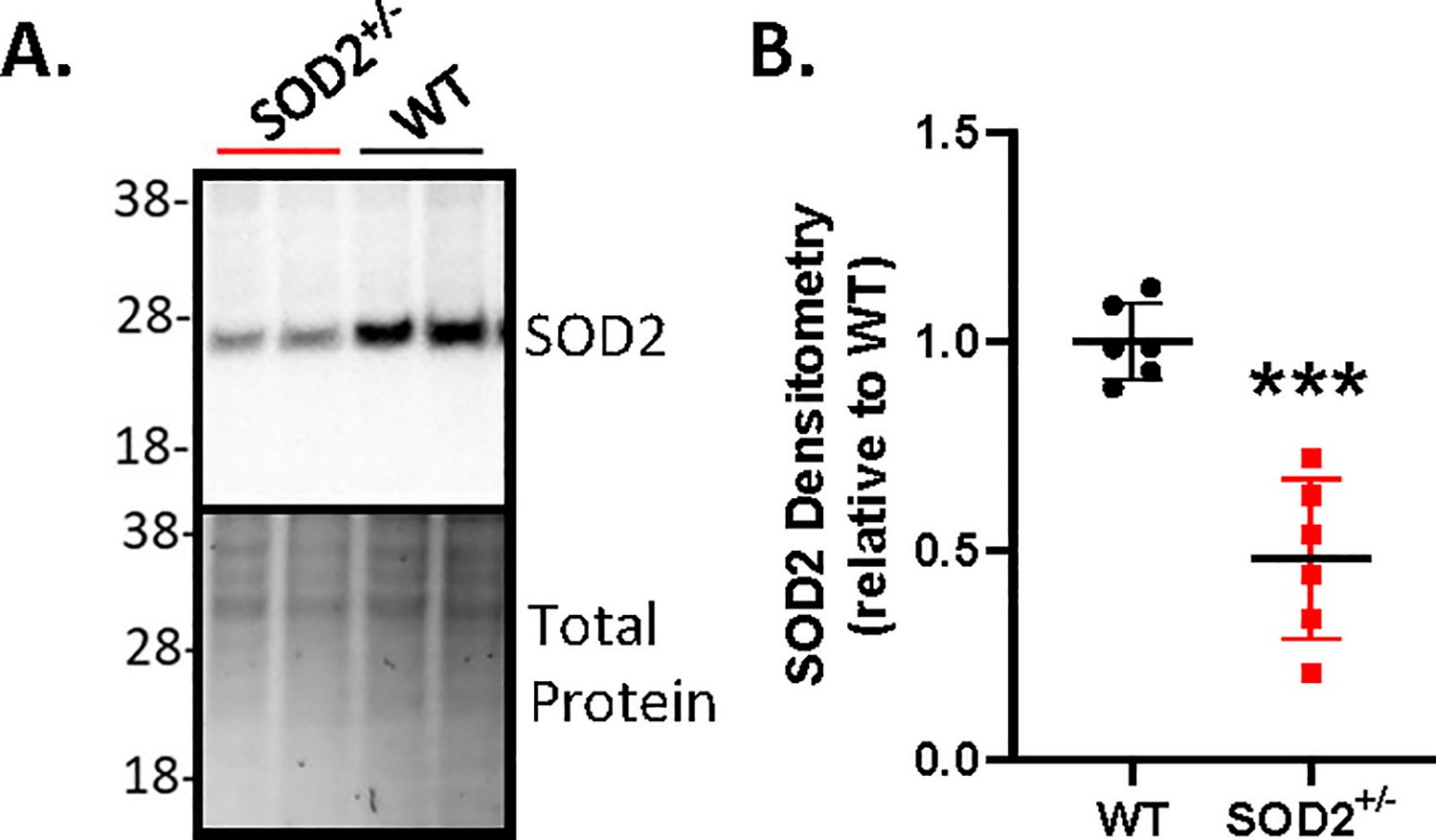
SOD^+/-^ mice have approximately half the brain SOD2 protein levels of WT mice. **A.** Example images of western blotting for SOD2 (upper panel) with Coomassie detection of total protein (lower panel) of brain tissue from 2 mice and **B.** quantification by densitometry of SOD2 protein levels in SOD2^+/-^ and WT mice. Each data point in B represents an individual mouse brain sample, ***p = 0.0005 (t-test with Welch’s correction).

As expected from previous reports, the SOD2^+/-^ mice developed normally, lived a normal lifespan compared with their WT littermates and did not display any overt pathology related to the reduced SOD2 level. Within the brain, the SOD2^+/-^ mice expressed approximately half the protein level of SOD2 that WT mice expressed (Fig 1).

### Reduced SOD2 does not influence electrophysiological responses following acute exposure to 22L, RML and ME7 prions

**Fig 2:**
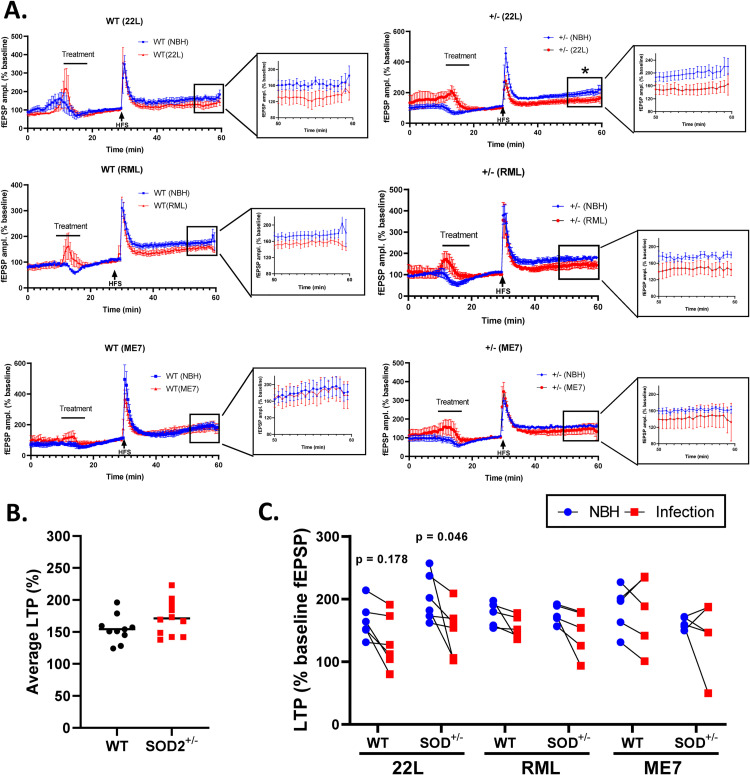
SOD2 reduction does not influence basal electrophysiology or response to acute challenge with prions. **A.** Example traces of long-term potentiation (LTP) in WT and SOD2+/- hippocampal slices following exposure to 22L, RML and ME7 prions. *p<0.05 (t-test with Welch’s correction of averaged LTP values). Traces show the mean and standard error of the mean of all slices (mice) per treatment (with magnification of the LTP measurement region. **B.** Average LTP of SOD2^+/-^ and WT brain slices relative to basal activity when incubated in artificial CSF (WT n = 10, SOD2^+/-^ n = 11). **C.** Average LTP, measured over 10 minutes and shown as percentage change from the pre-stimulation baseline readings, following addition of normal brain homogenate (NBH) or brain homogenate from terminally prion infected mice to brain slices in artificial CSF (22L: WT n = 6, SOD2^+/-^ n = 6; ME7: WT n = 5, SOD2^+/-^ n = 5; RML: WT n = 5, SOD2^+/-^ n = 5). Individual data points represent averaged electrode data from a single mouse, lines connect results indicating slices from the same mouse brain tested in parallel (analysis by Mann-Whitney comparing NBH LTP with infectious brain homogenate LTP).

Previous studies have shown that certain prion strains can cause acute synaptic toxicity, which is reflected in changes to neuronal long-term potentiation (LTP) [29]. To investigate whether the SOD2 reduction renders the SOD2^+/-^ mice more vulnerable to acute synaptotoxic insult by prions, we measured LTP in hippocampal slices. Fig 2A shows the combined traces from the brain slices of 12-week-old SOD2^+/-^ and WT mice exposed to one of the three strains, 22L, RML, or ME7 or control normal brain homogenate (NBH) in the artificial cerebral spinal fluid (CSF). For each mouse, slices were compared for their response to normal and infectious brain homogenate in parallel. Brain slices were assessed for LTP following 10 minutes exposure to the inoculum. No difference was seen in basal LTP between the genotypes in the artificial CSF (Fig 2B), indicating that the reduced SOD2 protein was not causing a basal level of neuroelectrophysiological dysfunction. In the kinetic curves, LTP is significantly reduced in response to the infectious brain homogenate compared with NBH for SOD2^+/-^ brain slices exposed to 22L (Fig 2A). When the average of the last 10-minute LTP was considered for each slice, the SOD2^+/-^ brain slices exposed to 22L remained the only significantly changed LTP response (Fig 2C). Overall, exposure to scrapie prions had limited significant effects on LTP for any strain in either WT or SOD2^+/-^ mice.

### SOD2^+/-^ mice do not have reduced lifespan when challenged with 22L, RML or ME7 prions compared with WT mice

**Fig 3:**
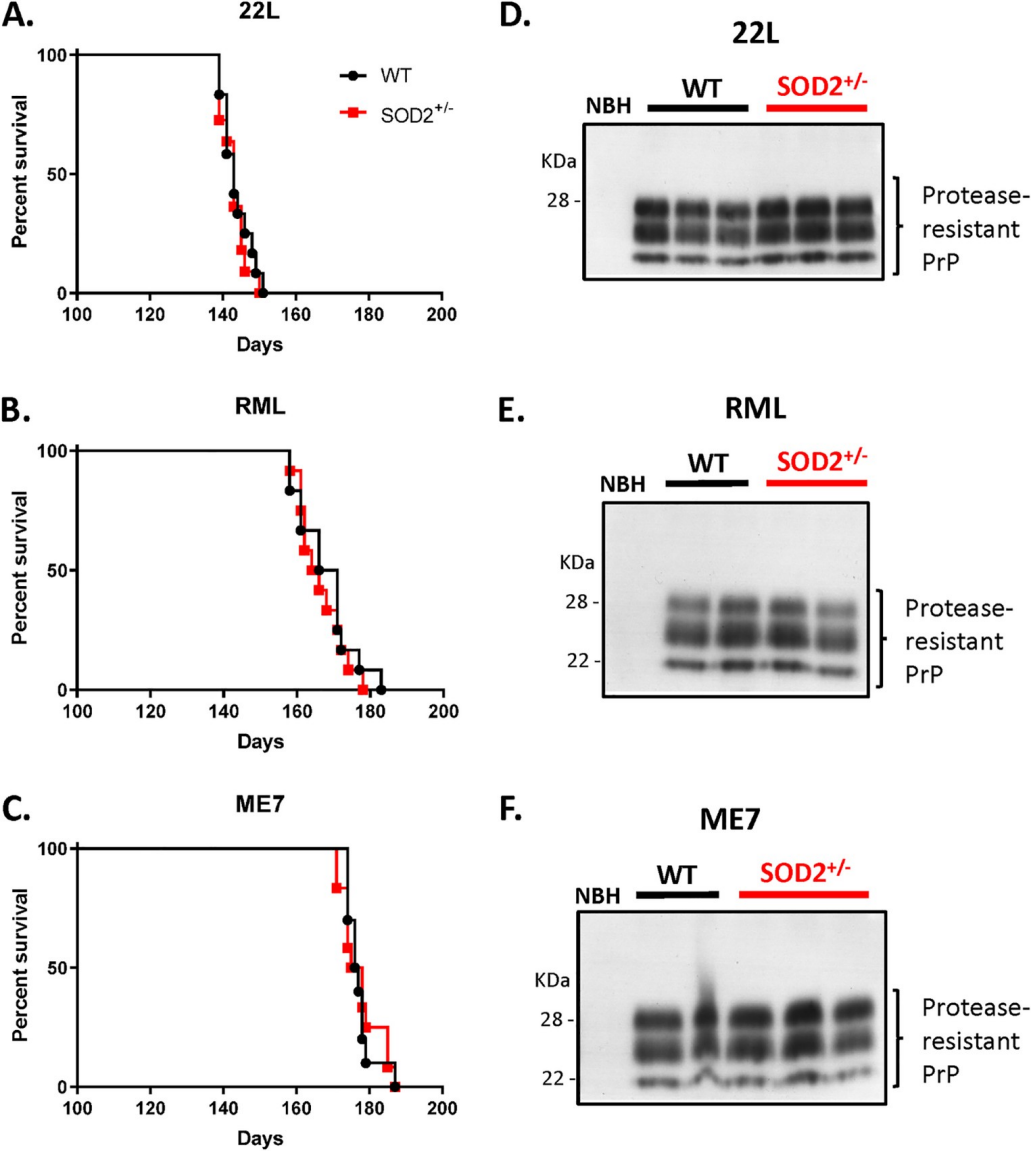
SOD2 reduction does not influence disease duration or protease-resistant PrP. Disease duration of mice inoculated with **A.** 22L (SOD2^+/-^ n = 11, WT n = 12), **B.** RML (SOD2^+/-^ n = 12, WT n = 12), and **C.** ME7 (SOD2^+/-^ n = 12, WT n = 10) prion strains. Representative western blotting for protease-resistant PrP in the **D.** 22L, **E.** RML, and **F.** ME7 infections. Each lane was loaded with equivalent amounts of proteinase K treated brain homogenate from an individual mouse.

We hypothesized that the lower protein levels of SOD2 in the SOD2^+/-^ mice would leave them vulnerable to increased oxidative damage during disease pathogenesis and, therefore, they might demonstrate more rapid disease progression to death. When inoculated with 22L, RML or ME7 prions, with the evaluator blinded to their genotype, the reduced SOD2 expression made no difference to the disease course (P = 0.59, 0.58 and 0.88 respectively; Fig 3A–3C). Furthermore, when the disease-associated PrP was examined by western blotting terminal brain tissue following protease digestion, accumulation was similar in both genotypes (Fig 3D–3F). Thus, reduced SOD2 was not influencing disease duration or the presence of disease-associated PrP.

### SOD2 protein levels are unchanged in SOD2^+/-^ prion disease brains

**Fig 4:**
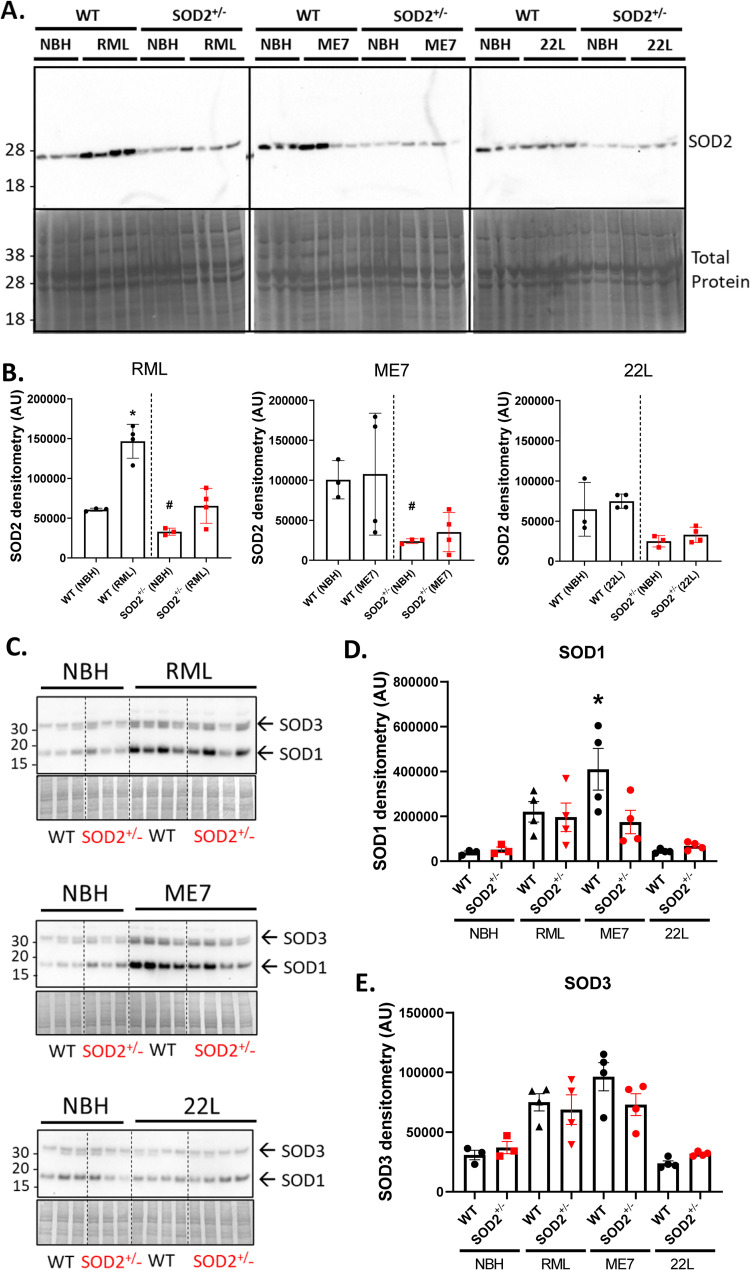
SOD2 protein expression remains low in SOD2^+/-^ mice brains collected at terminal prion infection. **A.** Western blotting of SOD2 (upper panels) and total protein Coomassie staining (lower panels) in the brains of SOD2^+/-^ and WT mice at terminal disease, each lane is an individual mouse brain. The NBH mice are the same in each blot. **B.** Densitometry of SOD2 bands; each point shows an individual mouse with error bars denoting the mean and standard deviation (NBH n = 3, infections n = 4). **p<0.01 different from the corresponding NBH control, #p<0.05 different from the corresponding WT condition (Kruskal Wallis). **C.** Western blots and quantification of **D.** SOD1 and **E.** SOD3 protein detection (NBH n = 3, infections n = 4). *P<0.05 different from the corresponding NBH control mice (Kruskal Wallis).

A possible explanation for the lack of difference across genotypes was that SOD2^+/-^ mice maintained the ability to up-regulate SOD2 protein to WT equivalent levels when under stress. If this were the case, at terminal disease all mice could have the same SOD2 protein levels and, consequently, there would be no difference in disease parameters. Western blotting brain tissue from terminal mice showed that SOD2 protein levels were not increased in prion infected SOD2^+/-^ mice relative to the NBH controls (Fig 4). Only the WT RML-infected mice showed a change in their SOD2 expression as a result of infection, with a significant ~2-fold increase over NBH mice, although the ME7-infected WT mice also showed highly variable SOD2 levels. It is unclear why SOD2 protein levels varied so widely in WT terminal disease mice across the strains, but this suggests the increase in the WT RML-infected mice has no relevance to pathogenesis and that fluctuations in SOD2 protein levels are unimportant for disease progression. We also considered protein levels of SOD1 and SOD3, finding only SOD1 significantly increased in the ME7 infected WT mice (Fig 4C–4E). No significant differences were apparent between genotypes suggesting that SOD1 and SOD3 are not changed as a compensatory response for the lower SOD2.

### SOD2+/- mice show no overt differences in brain pathology

**Fig 5:**
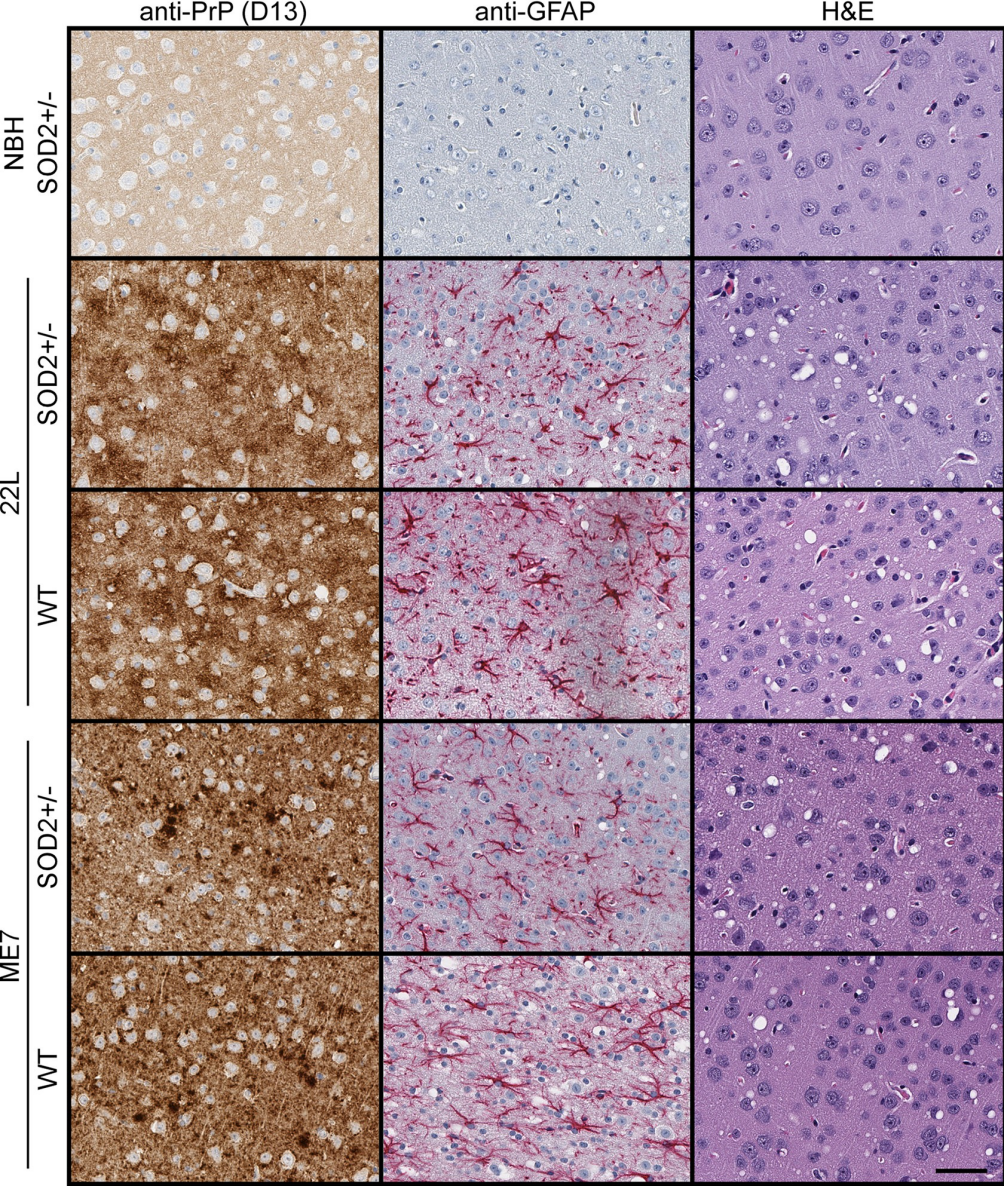
SOD2 reduction does not overtly influence disease pathology. Representative histological staining of PrP deposition, astrogliosis and spongiform change in the cerebral cortex of SOD2^+/-^ mice infected with 22L and ME7 strains compared with WT mice and the NBH controls. Scale bar = 50 μm.

As SOD2 knock-out mice kept alive post birth using anti-oxidant therapy develop a spongiform encephalopathy, we examined the PrD pathology present in the SOD2^+/-^ mice and WT mice infected with ME7 and 22L; representing the longest and shortest incubation periods of the strains tested respectively. No explicit difference was seen in PrP deposition, astrogliosis or spongiform change as a result of the reduced SOD2 (Fig 5).

### Lesion profiling confirms reduced SOD2 does not influence spongiform pathology

**Fig 6:**
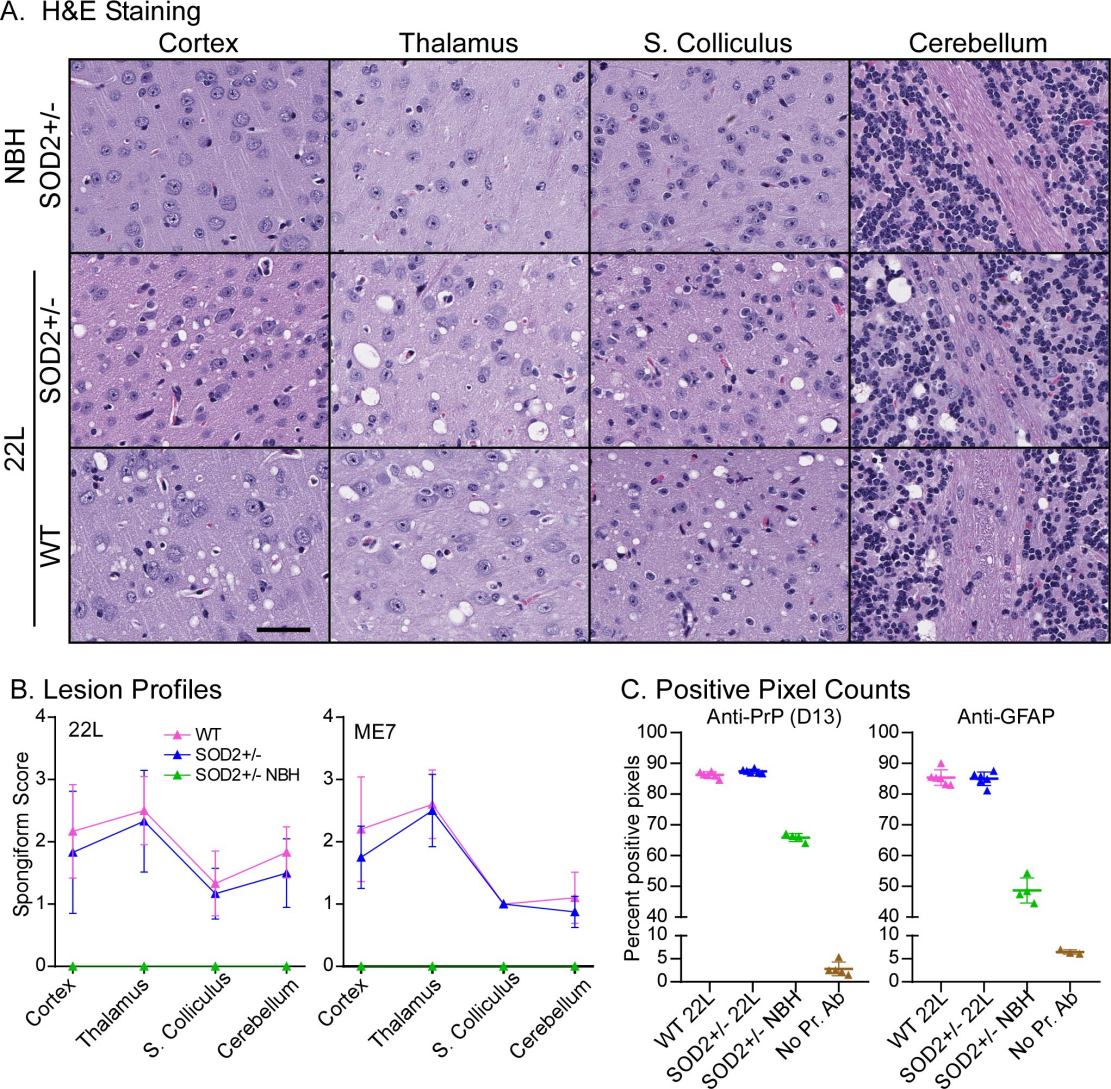
SOD2 reduction does not alter spongiform severity, or PrP or GFAP intensity. **A.** Representative images of four brain regions from 22L infected SOD2^+/-^ mice compared with WT and NBH controls. Scale bar = 50 μm. **B.** Lesion profiles of these four brain regions for 22L and ME7 infections showing the mean and standard deviation (NBH n = 4, infections n = 6). **C.** Quantification of PrP and GFAP antibody staining by positive pixel counts for the 22L infected mice (NBH n = 4, 22L n = 6, no primary antibody control n = 5). Each point is the count for an individual mouse with mean and standard deviation indicated.

To look closer at whether the reduced SOD2 might influence the severity of spongiform change in the SOD2^+/-^ mice, we examined four brain regions, grading their spongiform severity. The examiner was blinded to the mouse genotypes. The lesion profiles of the cerebral cortex, colliculi, thalamus and cerebellum were not different between the SOD2^+/-^ mice and WT controls (Fig 6A & 6B). This confirms the observation that reduction of SOD2 protein does not influence the development of spongiform pathology.

Additional pixel counts on the 22L infected brains also confirmed that PrP deposition and astrogliosis was not different between SOD2^+/-^ and WT mice. Overall, the reduction of SOD2 did not influence neuronal function upon challenge with infection, disease duration, or the appearance of the neuropathology and severity of the spongiform pathology across different brain regions in response to infection with different prion strains.

## Discussion

PrDs demonstrate increased oxidative damage within the brain that begins early in disease [4], and detection of SOD2 is changed in terminal brain tissue [1, 2, 10]. The development of a PrD-like spongiform encephalopathy in SOD2 knock-out mice, treated with antioxidants to delay their death from cardiomyopathy, indicated SOD2 may be a contributing factor to PrD pathogenesis. However, in mice expressing approximately 50% of the SOD2 protein present in the brains of wild-type animals, we saw no effect of the reduced SOD2 on neuronal function or disease pathogenesis upon trial with three prion strains. As SOD2 is essential for life, the lack of difference in lifespan of the PrD SOD2^+/-^ mice strongly indicates that SOD2 plays no role in the pathogenesis of PrD in mice.

In previous studies, a decrease in SOD2 gene expression [11] or protein [10] has been observed in animals infected with scrapie strains. In Bourgognon et al. [11], the infecting strain was RML; the same strain for which we observed an increase in protein levels of SOD2. The discrepancy may have arisen as Bourgognon et al. measured RNA levels rather than protein, as detected herein, or due to different genetic backgrounds of the mice. However, in human prion disease studies that examined protein levels in terminal brain tissue, increases in SOD2 protein were detected [1, 2], indicating that the increased SOD2 in the RML infections does mirror what occurs in human disease. In Park et al. [10], the strain studied was ME7 and the reduction in SOD2 measured by western blotting. We observed a highly variable response in SOD2 protein detection for the WT ME7 infected mice, with two of four brains showing a decrease. The variable SOD2 detection in our ME7 infections and the genotypes of this cohort were re-checked and a reason for the variability was not apparent. Since the WT protein intensities of SOD2 were variable across all infections and neither WT SOD2 protein levels nor the reduced levels in the SOD2^+/-^ mice influenced the outcome of the disease, fluctuations of SOD2 protein levels appear to be a secondary effect of the disease process.

We also observed discrepancies between our current LTP data and previously published electrophysiology studies looking at prion-induced synaptotoxicity where, in contrast with the previously published studies, we found few significant changes [29, 30]. These differences likely arise due to the different strains examined. The original studies used the mouse-adapted human M1000 strain, whereas in the current study we considered mouse-adapted scrapie strains. Differences in the aggressiveness of different prion strains and regions of the brain attacked are widely known. Even herein we found that the different strains produced different responses in the WT mice with a significant increase in SOD2 protein only in RML infected mice and an increase in SOD1 only in ME7 infected mice. The SOD2^+/-^ mice showed the only significant change in LTP when challenged with 22L inoculum, possibly because this is the most aggressive strain we looked at with the shortest time to death. WT mice challenged with the same dosage of M1000 inoculum as we used here die around the same time as those challenged with 22L, although the timeframe is narrowed (145 dpi +/- 2 days for M1000 [4, 12] vs 145 dpi +/- 6 days for 22L herein). Therefore, an explanation for the discrepancy with our WT data is that 0.5% w/v does not contain enough scrapie prions to induce toxicity but 22L, causing the most aggressive disease, is closest to a toxicity threshold surpassed by the mouse-adapted human prions. The difference between the WT and SOD2^+/-^ LTP in response to 22L was not sufficiently great that we believe it to be important to the disease process, especially when considered in the context of the lack of change in survival time or pathology.

That the SOD2 protein levels were only increased in the RML infected and the SOD1 protein levels only increased in the ME7 infected WT mice is interesting. This raises the question of whether some strains cause greater oxidative stress than other strains or influence different redox producing/detoxifying pathways. Whilst there is ample evidence for oxidative stress in the brains of PrD mice infected with multiple strains [2, 4, 9, 13, 31, 32], we are not aware of any studies that have found ROS levels to be higher in in one strain over another. Comparison of indirect markers of oxidative stress across several strains demonstrated no difference in the detection of these markers [31]. More investigation may be required to understand if the strains do cause differing levels of stress and, if so, whether this is important to disease pathogenesis.

While we showed no changes in the other SOD family members in the SOD2^+/-^ mice to suggest the deficit was annulled by compensation mechanisms, this is not to say none were occurring. Potentially other redox balancing systems, outside the SOD family, may be providing some alleviation of increased ROS and PrP itself has been linked with protection against oxidative stress [33–36]. Heightened ROS is associated with increased processing of PrP at the beta-cleavage site and during infection this becomes the dominant cleavage event [37–39]. Beta-cleavage and especially the released N-terminal fragment, N2, that is not part of the protease resistant core, have been shown to have protective properties against oxidative stress [40–42]. The N2 fragment has additionally be shown to localize to the mitochondria when administered to cells in their media [42], possibly effecting a protective response within the organelle. Therefore, many other cellular mechanisms may render the reduction of SOD2 redundant for survival or pathology during PrD.

Our data indicates that SOD2 is not involved in PrD pathogenesis but this does not mean that other redox pathways and cellular antioxidant molecules or proteins are likewise unimportant. Indeed, SOD1 knock-out does reduce the lifespan of prion infected mice [5] and, conversely, knock-out of the oxidant protein NADPH oxidase-2 (NOX2) was found to increase survival time and delay symptom onset [43]. PrP has also been functionally linked to several redox signaling pathways, including NOX2 and neuronal nitric oxide synthase [10, 44–47] and any combination of these may become dys-regulated during disease leading to oxidative damage. Furthermore, a number of anti-oxidant based therapies have been found to extend survival time in mice, including the administration of a SOD2 mimetic in infectious disease [12] and a combination therapy using a nanoformulation of pomegranate seed oil with neural progenitor transplantation in a genetic disease model [48]. While SOD2 may contribute to the failings of these pathways in PrD, ultimately it appears that oxidative damage caused by other failing pathways is of greater significance to the development of disease pathology.

A clear limitation of our study is that translation to humans is not necessarily guaranteed. For example, SOD1 knock-out mice have no overt phenotype up to six-months of age but develop age-dependent muscle atrophy later in life [49, 50]. However, human patients with mutations resulting in minimal SOD1 activity demonstrate signs of the deficit from around 6–9 months old with severe loss of motor functions in early childhood [51, 52]. Thus, the SOD1 knock-out mouse model does not fully recapitulate human SOD1 deficiency. However, a reported genetic SOD2 deficiency patient presented similarly to what was observed in SOD2 knockout mice, with death occurring a few days post birth [53]. The causative mutation was recessive and the parents of the patient, who were heterozygous, showed no signs of the deficiency when just one functional allele was present. Consequently, the SOD2 knock-out model might provide a close indication of how reduced SOD2 protein influences human biology in the context of PrD, but this cannot be concluded without investigation within a human model of disease.

We cannot exclude the possibility that a heterozygous reduction of SOD2 is insufficient to see any changes. While this is possible, and another heterozygous mouse experiment examining cysteine string protein alpha also showed no difference in incubation period [54], if the observed variations in SOD2 protein expression levels was a critical factor in PrD pathogenesis it is likely that some pathological effect would have been observed in the SOD2^+/-^ mice. We conclude that reduction of SOD2 does not alter PrD pathogenesis and changes in SOD2 protein levels are likely a down-stream effect of disease rather than causative of damage.

## Supporting information

S1 Raw images (PDF)
