# Correction: Reduced SOD2 expression does not influence prion disease course or pathology in mice

**DOI:** 10.1371/journal.pone.0360777

**Published:** 2026-10-19

**Authors:** Simote T. Foliaki, Brent Race, Katie Williams, Chase Baune, Bradley R. Groveman, Cathryn L. Haigh

In Fig 5, the 22L SOD2^+/-^ and 22L WT rows are switched. The corrected Fig 5 is provided with this notice, and the image data provided in support of Figs 5 and 6 are provided in S1 and S2 Files.

**Fig 5:**
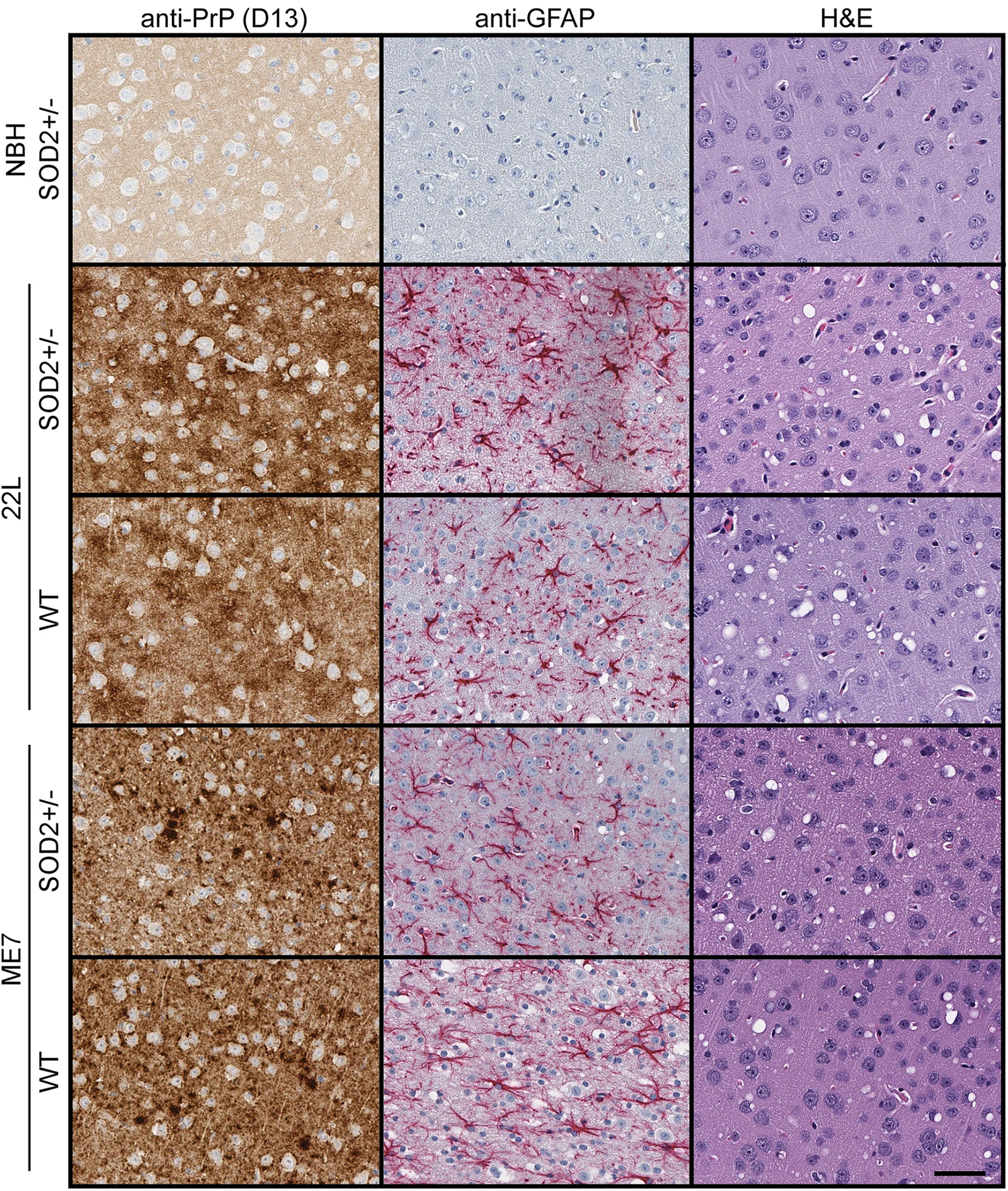
SOD2 reduction does not overtly influence disease pathology. Representative histological staining of PrP deposition, astrogliosis and spongiform change in the cerebral cortex of SOD2^+/-^ mice infected with 22L and ME7 strains compared with WT mice and the NBH controls. Scale bar = 50 μm.

## Supporting information

S1 File Original image data underlying Fig 5. (ZIP)

S2 File Original image data underlying Fig 6. (ZIP)
