# Supplementary material for: Reduced SOD2 expression does not influence prion disease course or pathology in mice
Source: PLoS One. 2021 Nov 4;16(11):e0259597. doi: 10.1371/journal.pone.0259597 (PMC8568125; doi:10.1371/journal.pone.0259597)
Supplement: S1 Raw images — (PDF) [file pone.0259597.s001.pdf]

Simote T Foliaki, Brent Race, Katie Williams, Chase Baune, Bradley R Groveman, Cathryn L Haigh. **Reduced SOD2 expression does not influence prion disease course or pathology in mice**

Supplementary File 1: *Un-cropped blot images.* The un-cropped blot files are shown by figure below. Blots were generated and images captured as described in the methods section of the main paper.

Figure 1

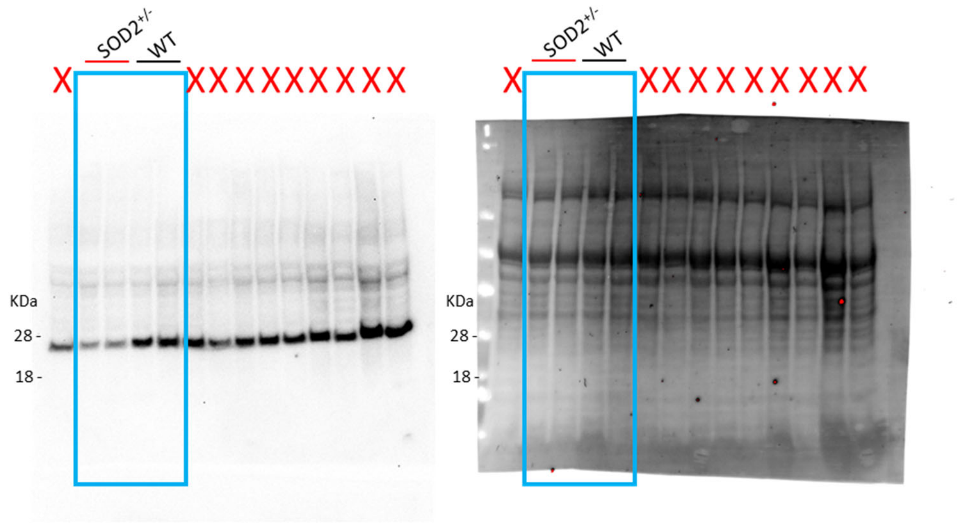

Figure 2

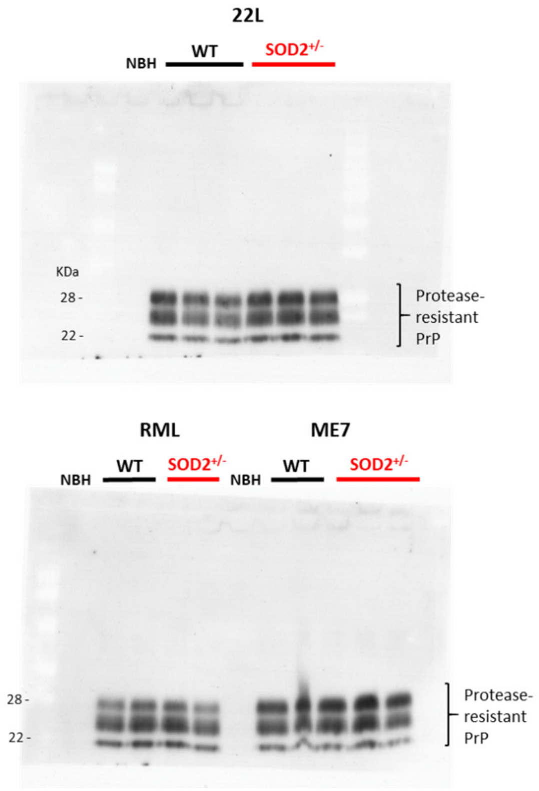

Figure 3

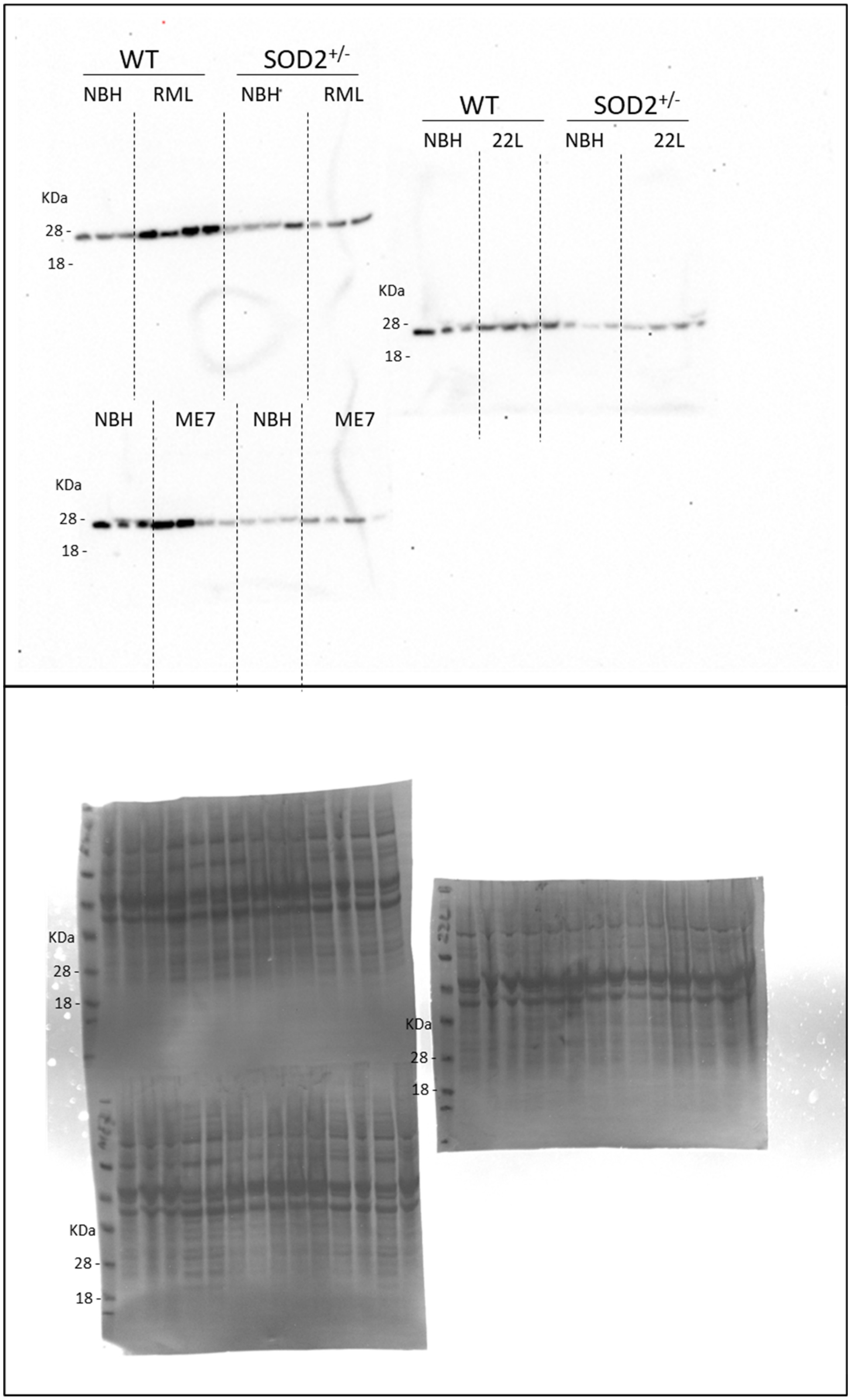
WT
SOD2+/-
NBH
RML
NBH
RML
WT
SOD2+/-
NBH
22L
NBH
22L
KDa
28 -
18 -
NBH
ME7
NBH
ME7

Figure 4

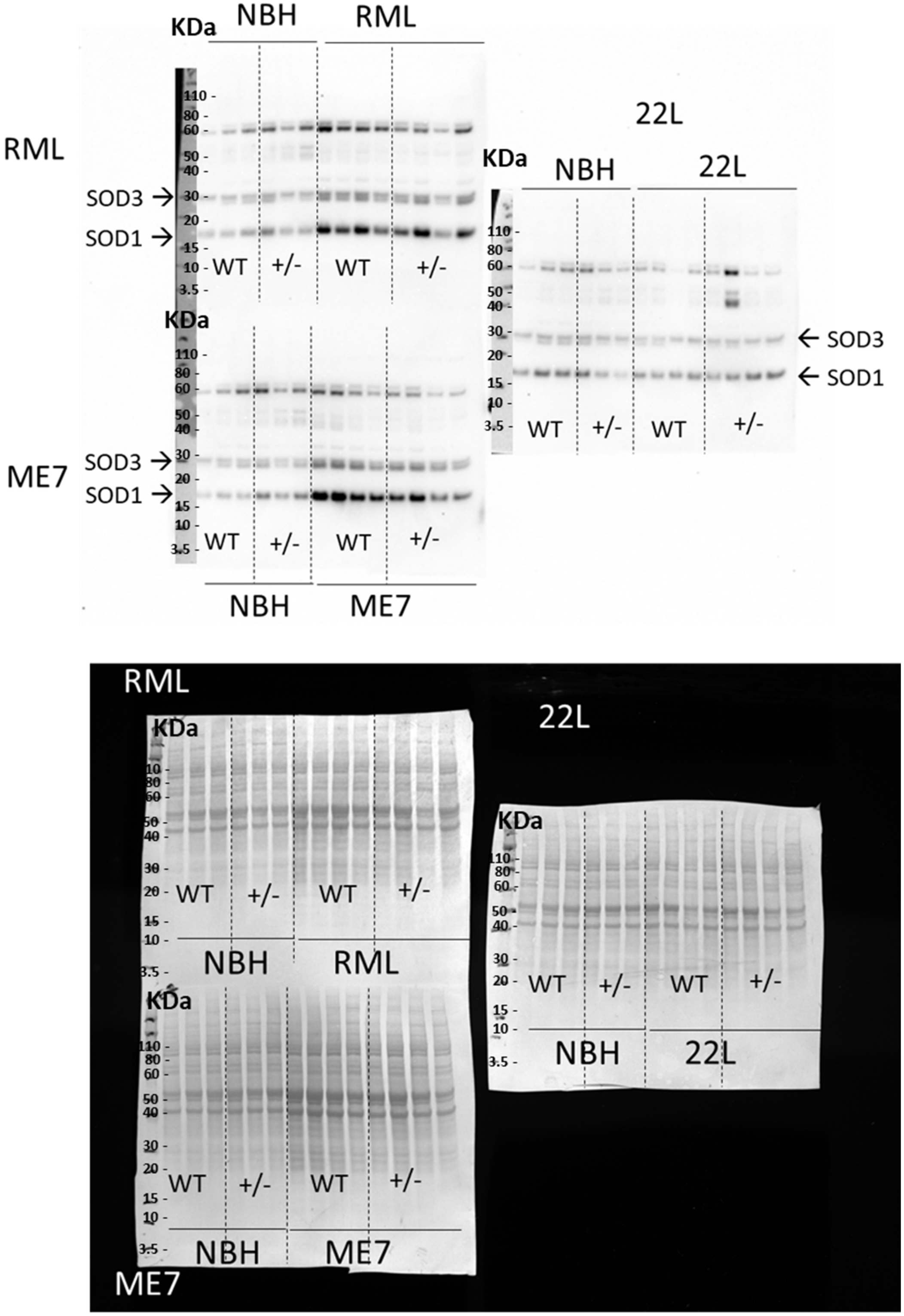
RML
KDa
NBH
RML
110
80
60
50
40
30
20
15
10
3.5
SOD3 →
SOD1 →
WT
+/-
WT
+/-
ME7
SOD3 →
SOD1 →
NBH
ME7
22L
KDa
NBH
22L
← SOD3
← SOD1
WT
+/-
WT
+/-
